# *Bacillus subtilis* KLBMPGC81 suppresses appressorium-mediated plant infection by altering the cell wall integrity signaling pathway and multiple cell biological processes in *Magnaporthe oryzae*

**DOI:** 10.3389/fcimb.2022.983757

**Published:** 2022-09-09

**Authors:** Lianwei Li, Yanru Li, Kailun Lu, Rangrang Chen, Jihong Jiang

**Affiliations:** The Key Laboratory of Biotechnology for Medicinal Plants of Jiangsu Province, School of Life Science, Jiangsu Normal University, Xuzhou, China

**Keywords:** *magnaporthe oryzae*, appressorium, plant infection, cell wall integrity, autophagy, cell cycle, biocontrol, *bacillus subtilis*

## Abstract

*Magnaporthe oryzae* is one of the most destructive crop pathogens in the world, causing huge losses in rice harvest every year. *Bacillus subtilis* is a potential biocontrol agent that has been explored in many crop systems because it is a potent producer of bioactive compounds. However, the mechanisms by which these agents control rice blasts are not fully understood. We show that *B. subtilis* KLBMPGC81 (KC81) and its supernatant (SUP) have high antimicrobial activity against *M. oryzae* strain Guy11. To better exploit KC81 as a biocontrol agent, the mechanism by which KC81 suppresses rice blast pathogens was investigated. This study shows that KC81 SUP is effective in controlling rice blast disease. The SUP has a significant effect on suppressing the growth of *M. oryzae* and appressorium-mediated plant infection. KC81 SUP compromises cell wall integrity, microtubules and actin cytoskeleton, mitosis, and autophagy, all of which are required for *M. oryzae* growth, appressorium development, and host infection. We further show that the SUP reduces the activity of the cyclin-dependent kinase Cdc2 by enhancing the phosphorylation of Cdc2 Tyr 15, thereby impairing mitosis in *M. oryzae* cells. SUP induces the cell wall sensor MoWsc1 to activate the cell wall integrity pathway and Mps1 and Pmk1 mitogen-activated protein kinases. Taken together, our findings reveal that KC81 is an effective fungicide that suppresses *M*. *oryzae* growth, appressorium formation, and host infection by abnormally activating the cell wall integrity pathway, disrupting the cytoskeleton, mitosis, and autophagy.

## Introduction

*Magnaporthe oryzae*, the causative pathogen of rice blast, causes extensive losses in rice cultivation worldwide (Dagdas et al., 2012). No cure has been found for this condition, and indiscriminate use of chemical control agents may result in pathogen resistance and other environmental and health issues (Karthikeyan and Gnanamanickam, 2008; Spence et al., 2014). To produce food in a sustainable and environmentally friendly way, the biological control of crop diseases is currently receiving greater attention because of its high specific activity against target pathogens, good adaptability to the environment, synergy with crops, and multifunctional working mechanisms. Microbial resources with antifungal properties have been investigated as potential biocontrol agents in a variety of agricultural systems. Among the microbial candidates for biocontrol, *Bacillus* spp. are considered one of the most valuable because they produce potent bioactive compounds with diverse biological properties, and their sporulating properties allow them to survive in harsh environments.

*Bacillus* spp. can produce many metabolites and destroy the cells of plant pathogenic fungi. *Bacillus cereus* HS24 prevents extracellular calcium from entering *M. oryzae* conidia and significantly reduces the concentration of free intracellular Ca^2+^, thereby inhibiting conidial germination (Huang et al., 2020). *Bacillus licheniformis* BL06-SP reduces chitin content, inhibits appressorium formation, and attenuates the pathogenicity of *M. oryzae* (Liu et al., 2021). *Bacillus safensis* B21 inhibits the hyphal growth of *M. oryzae* by producing iturin, which can change hyphal membrane permeability (Rong et al., 2020). *Bacillus* sp. CS30 can substantially inhibit *M. oryzae* growth, and its metabolite surfactin can induce the accumulation of ROS and *M. oryzae* cell death, thereby reducing *M. oryzae* pathogenicity in plants (Wu et al., 2019). The marine bacterium *Bacillus subtilis* BS155 can inhibit *M. oryzae* growth by producing the antifungal compound fengycin BS155, which can induce membrane damage, the dysfunction of organelles, mitochondrial membrane destruction, oxidative stress, and chromatin condensation, leading to the death of *M. oryzae* mycelial cells (Zhang and Sun, 2018). In all these reports, however, the observations do not clearly reveal *Bacillus* spp.’s mode of action or its pleiotropic effects on cell viability. Therefore, this study aims at a more comprehensive investigation of how *Bacillus* spp. affect fungal viability to determine its efficacy as a novel fungicide. The poisoning process of *M. oryzae* by *Bacillus* spp.’s secondary metabolites and its response to this poisoning also require further study.

The response of fungi to external stress is regulated by a variety of signaling pathways and biological processes, such as the cell wall integrity signaling pathway (CWI pathway), cell cycle, autophagy, and cytoskeleton assembly. The fungal cell wall is a complex structure that serves as a protective barrier against environmental stress (Latge, 2007). Cell wall damage is detected by cell wall stress sensors located on the plasma membrane, and especially by the cell wall stress response component Wsc1 involved in downstream signaling pathways (Bermejo et al., 2010; Kock et al., 2016). The CWI pathway responds and adapts to diverse environmental conditions in the budding yeast *Saccharomyces cerevisiae* and pathogenic fungi, such as *M. oryzae* (Levin, 2011; Yin et al., 2016). In *M*. *oryzae*, the CWI pathway mainly consists of a conserved mitogen-activated protein (MAP) kinase pathway. MAP kinase pathways play an essential role in mycelial morphogenesis, response to extracellular stresses, appressorium formation, and virulence (Xu et al., 1998; Jeon et al., 2008; Yin et al., 2016). The disruption of MAP pathway components leads to cell lysis (Xu et al., 1998; Levin, 2011; Yin et al., 2016). In response to internal cues and environmental challenges, MAPK MoMps1 and MAPK MoPmk1 are activated *via* protein phosphorylation. Consequently, MoMps1 and MoPmk1 phosphorylate transcription factors to regulate the nuclear expression of genes involved in cell wall biosynthesis, cell cycle progression, autophagy, appressorium formation, appressorium penetration, and invasive growth (Xu et al., 1998; Jeon et al., 2008; Yin et al., 2016; Yin et al., 2020; Feng et al., 2021; Oses-Ruiz et al., 2021).

Reasonable regulation of the cell cycle in fungi is an effective way to deal with damage and environmental stress (Shiozaki and Russell, 1995; Petersen and Hagan, 2005; George et al., 2007; Cansado et al., 2022). Cell cycle control is a crucial biological process underlying growth, development, and survival (Kagami and Yoshida, 2016). Cell cycle entry is controlled by cyclin-dependent kinase (CDK) Cdc2/Cdc28 activity (Mendenhall and Hodge, 1998; Petersen and Hagan, 2005). Inhibitory phosphorylation of the well-conserved Tyr15 of Cdc2 is known to affect CDK activation (Wang et al., 2004; Sgarlata and Perez-Martin, 2005; Liu et al., 2015). In addition to binding to cyclins, full activation of Cdc28 requires phosphorylation by Cak1 kinase at conserved T residues in the T loop (Liu and Kipreos, 2000; Liu et al., 2015).

Autophagy, a stress response, plays a key role in the survival of eukaryotes (Kroemer et al., 2010; Mizushima and Komatsu, 2011). Autophagy is a degradation process in which cells break down their components, recycle macromolecules, and provide energy essential for cell survival under various stressors (Yin et al., 2020; Wang et al., 2021). In addition, the cell cycle is necessary for infection-associated autophagy (Veneault-Fourrey et al., 2006). The cytoskeleton is a dynamic network critical for various cellular processes, including the cell cycle, vesicle trafficking, pathogenicity, and cell signaling in response to biotic and abiotic stimuli in *M. oryzae* (Saunders et al., 2010; Dagdas et al., 2012; Li et al., 2017).

These signaling pathways and cellular processes are involved in the response to extracellular stress and pathogenicity of *M. oryzae*, but whether they are involved in the interaction with antifungal biocontrol bacteria has not been systematically studied. In other words, the effects of *B. subtilis* on these processes that are closely related to the growth, development, and pathogenicity of *M. oryzae* remain unclear. In this study, we explored the effect of the supernatant (SUP) of *B. subtilis* KC81 on the growth and infection-related development of *M. oryzae.* KC81 SUP showed a strong antimicrobial effect on *M. oryzae*. We were interested in understanding how it acts on cellular processes necessary for plant infection. We provide evidence that the SUP acts as a fungicide on *M. oryzae*. It disturbs cell wall integrity, cell cycle progression, cytoskeleton, and autophagy and prevents appressorium-mediated plant infection. Importantly, we show that the cell wall integrity pathway, autophagy, and cell cycle activity are essential components for *M. oryzae* to mount a concerted stress response to KC81 SUP. This study not only provides valuable information on how *B. subtilis* suppresses *M. oryzae* but also sheds light on how pathogenic fungi mount a concerted stress response to external biocontrol agents. This is conducive to the further application of biocontrol bacteria to undermine pathogenic fungi’s adaptability and survival.

## Materials and methods

### Strains and culture conditions

*M. oryzae* strain Guy11 (Li et al., 2017) was used as the wild type. KC81 (Li et al., 2018) was used as the biocontrol bacterium. KC81 was inoculated on Luria-Bertani (LB) agar plates at 37°C. Liquid LB medium was used to prepare the SUP liquid. *M. oryzae* Guy11 strains were maintained in complete medium (CM) plates at 28°C. For conidiation, mycelial blocks were maintained on SDC (100 g of straw, 40 g of corn powder, 15 g of agar in 1 L of distilled water) media at 28°C for 7 days in the dark, followed by 3 days of continuous illumination under fluorescent light (Zhang et al., 2011).

### Co-culture assays of *B. subtilis* and *M. oryzae* Guy11

The dual culture method was used to evaluate the antifungal activity of KC81. Cultures were grown in LB broth and shaken at 200 rpm for 12 h in a 37°C incubator. A culture or LB broth (control) was placed in a straight line on one side of the CM plate, and 2 mm *M. oryzae* hyphal agar plugs were placed on the other side of the CM plate 2 cm away from the line. After 7 days at 28°C, the growth of *M. oryzae* Guy11 was observed.

KC81 SUP was obtained by centrifugation and filtration through a 0.22 µm biofilter when the OD_600_ values reached 1.0–1.2. The cell-free SUP or LB medium (control) was mixed with CM medium at a ratio of 1:6 to prepare cm plates. The *M. oryzae* strain Guy11 was inoculated into the middle of the plate and cultured at 28°C for 7 days.

### Exposure of *M. oryzae* to *B. subtilis* SUP during plant infection

Four treatments of two-week-old rice seedlings (*Oryza sativa* cv. CO39) were sprayed with 5 mL of conidial suspension (5 × 10^4^ spores/mL) and kept in a growth chamber at 28°C with 90% humidity. They were kept in the dark for the first 24 h, followed by a 12 h light/12 h dark cycle. The treatments included: (1) rice seedlings spray-incubated with strain Guy11; (2) 5 mL SUP sprayed onto rice seedlings 1 day after the strain Guy11 spray treatment (SUP, 1 day later); (3) rice seedlings spray-incubated with strain Guy11 1 day after spray treatment with 5 mL SUP (SUP, 1 day before); (4) rice seedlings sprayed with strain Guy11 mixed with 5 mL SUP (SUPM). The leaf lesions were photographed after 7 days.

### Infection process observation

To study the effect of KC81 on infection, the SUP of crude lysate was used. Approximately 1 g of wet cells were suspended in 10 mL of 10 mM phosphate-buffered saline (PBS) pH7.2. After sonication on ice for 0.5 h, the crude lysate was centrifuged at 12000 rpm for 10 min to remove cellular debris, and SUP was stored at −80°C until used for analysis. The spore suspension and SUP were mixed in a 1:1 ratio to make the concentration of the conidial suspension 5 × 10^5^ spores/mL. Ten μL of the mixture of *M. oryzae* conidial suspension and the supernatant of crude enzyme solution (v\v=1:1) was dropped on the surface of the rice leaves growing for approximately 10 d, and cultured at 28°C in the dark for 24 h. The formation of the appressorium was observed under a microscope and photographed. A mixture of *M. oryzae* conidial suspension and the SUP of crude lysate at a concentration of 5 × 10^4^ spores/mL was dropped on the lower epidermis of barley leaves and then incubated in a humid and dark room at 28°C. Microscopic observations were performed 24 h after the inoculation.

### Fluorescence observation

Either the mycelia blocks of Guy11 or Guy11 with different fluorescent markers were inoculated into a conical flask of 75 mL CM medium and oscillated (160 rpm) at 28°C for 24 h. Then, 25 mL of the cell-free supernatant, with a concentration of cultures with OD_600_ values of 1.0–1.2, was added to the treatment group. Mycelial growth was observed at different times under a microscope.

For the fluorescence observation of appressorium development, 20 μL of the mixture of the crude lysate SUP and conidia suspension (v\v=1:1) was dropped onto the surface of the hydrophobic glass coverslips and incubated at 28°C to induce appressorium formation. The germination tubes and appressoria were intermittently observed under a fluorescence microscope (Olympus DM5000B) after 2, 4, 6, 8, and 24 h of incubation.

Calcofluor white (CFW) staining was performed using fluorescent brightener 28 (10 μg/mL; Sigma-Aldrich) for hyphal microscopy.

### Western blot analysis

The mycelia block of Guy11-GFP-Atg8 was inoculated into a conical flask of 75 mL CM medium and oscillated (160 rpm) at 28°C for 24 h. Then, a third volume of the cell-free supernatant with a concentration of cultures OD_600 =_ 1 was added as the treatment group. Approximately 150–200 mg of mycelia were ground into powder in liquid nitrogen and resuspended in 1 mL of extraction buffer (10 mM Tris-HCl [pH 7.5], 150 mM NaCl, 0.5 mM EDTA, 0.5% NP40), to which 1mM PMSF was freshly added. The total protein was separated on a 12% SDS-PAGE gel and transferred to a polyvinylidene fluoride (PVDF) membrane. The membrane was analyzed by western blotting, using anti-GFP antibodies.

For the detection of Cdc2 phosphorylation, the Guy11 protein with the supernatant treatment was extracted, as previously described, by resuspending in 1 mL of extraction buffer and 1 mM PMSF, 10 μL of protease inhibitor cocktail, and 10 μL of phosphatase inhibitor cocktail were freshly added. A Cdc2 antibody (Cell Signaling Technology, #77055, USA) was used to detect endogenous Cdc2 expression. Phospho-Cdc2 (Tyr15) and phospho-cdc2 (Thr161) antibodies (Cell Signaling Technology, #9111 and 9114, respectively, USA) were used to detect phosphorylated Cdc2.

### Assays for appressorium formation and statistical analysis

The SUP of the crude lysate was used to form the appressorium. The spore suspension and SUP were mixed 1:1 to make the concentration of conidial suspension 5 × 10^5^ spores/mL, and 20 µL of the mixed conidial suspension was dropped onto a hydrophobic glass coverslip and incubated at 28°C with moisture. A conidial suspension of rice blast fungus without SUP was used as a control. Conidial germination and appressorium formation were observed at 2, 4, 6, and 24 h. One hundred conidia were assessed for each treatment, and each treatment consisted of three replicates. This experiment was repeated three times.

All experiments were repeated three times. Error bars represent standard deviation (SD), and values with asterisks represent significant differences (*p* < 0.01). Representative data were analyzed using SPSS software (version 20.0; IBM Corporation) using a one-way analysis of variance (ANOVA).

## Results

### KC81 SUP reduces the ability of *M. oryzae* to cause rice blast disease

**Figure 1:**
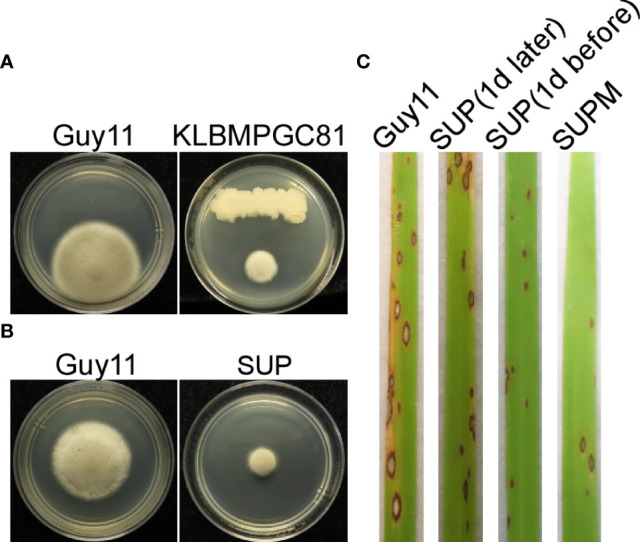
Biocontrol of *M. oryzae* using *B subtilis* KC81. **(A)** Cultured strains Guy11 on CM plates with *B subtilis* KC81. **(B)** Cultured strains Guy11 on CM plates with KC81 SUP. **(C)** Leaves showing blast symptoms in the presence of KC81 SUP. All rice seedlings were treated with strain Guy11. Guy11: *M. oryzae*; SUP: cell-free culture supernatants of strain KC81; SUP (1 day later) and SUP (1 day before): SUP sprayed onto rice seedlings 1 day after or 1 day before the strain Guy11 spray treatments, respectively; SUPM: rice seedlings sprayed with strain Guy11 mixed with SUP.

The ability of KC81 to inhibit *M. oryzae* Guy11 growth was evaluated using an antagonistic experiment. KC81 and its SUP were shown to exhibit strong activity against *M. oryzae* Guy11 (**Figures 1A, B**). Since *B. subtilis* can produce many secondary metabolites against pathogenic fungi, KC81 SUP was tested to determine its potential to control rice blast disease. SUP exhibited prominent biocontrol efficacy in rice seedling infection assays. Seven days after inoculation, a significant reduction in the number of lesions, varying in spot size, was observed. We also found that SUP showed protective and curative effects on rice against strain Guy11, and when a SUP was sprayed in combination with the Guy11 conidia (SUPM), the highest biological control efficiency and healthy plant growth were observed (**Figure 1C**).

### KC81 SUP impairs appressorium development and function in *M. oryzae*

**Figure 2:**
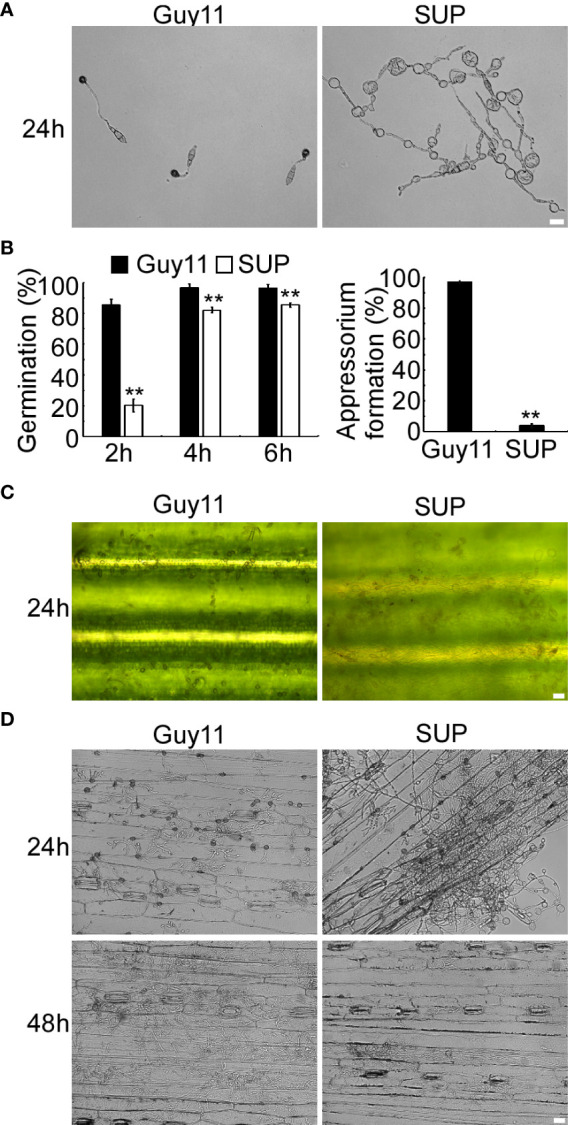
KC81 SUP impairs appressorium development and function of *M. oryzae*. **(A)** Appressorium formation assay on hydrophobic surfaces. Conidia were incubated on hydrophobic surfaces and the samples were observed. **(B)** Statistical analysis of spore germination rates and appressorium formation rates at different time points. The percentage at a given time was recorded by observing at least 100 conidia for each strain and the experiment was repeated thrice. Error bars represent SD and two asterisks represent significant differences (*p* < 0.01). **(C)** Appressorium formation assay on rice leaves. Conidia were incubated on rice leaves and the samples were observed after 24 (h) **(D)** Microscopic observation of infectious growth on barley. Excised barley leaves from 7-day-old barley seedlings were inoculated with conidial suspension (5 × 10^4^ spores/mL). Infectious growth was observed at 24 h and 48 h post-inoculation. Bar = 20 μm.

KC81 SUP reduced the frequency of appressorium differentiation when applied to ungerminated *M. oryzae* conidia (**Figure 2**). The differences in conidial germination rate between the SUP-treated and control treatments diminished after 4 h, but after 24 h of treatment with SUP, only 4% conidia formed appressoria. The appressorium formation rate of the treated group was significantly lower than that of the control group (97%) (**Figures 2A, B**). The conidia of the treated group developed long germ tubes with branches and abnormally swollen cells, rather than the appressorium (**Figure 2A**). Similar results were observed on rice leaves; conidia exposed to SUP only developed very small numbers of appressoria (**Figure 2C**). Infection assays on barley epidermal cells also showed that only few conidia exposed to SUP successfully invaded host cells compared with the control (**Figure 2D**). These results indicate that KC81 SUP inhibits appressorium formation and invasion development in *M. oryzae*.

### KC81 SUP weakens cell walls of *M. oryzae*

**Figure 3:**
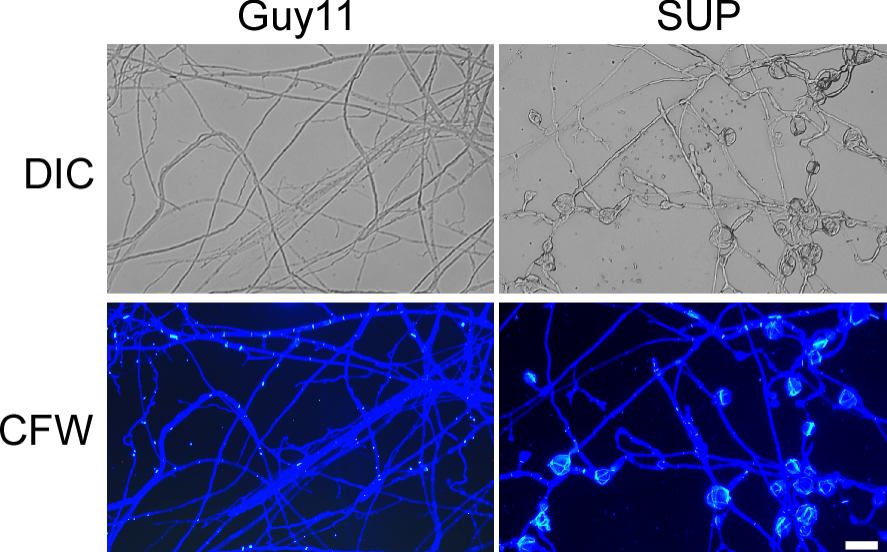
KC81 SUP destroys cell wall integrity of *M. oryzae*. KC81 SUP altered the distribution of chitin in the cell wall of *M. oryzae*. Hyphae were stained with 10 μg/mL calcofluor white (CFW) for 5 min without light before being photographed. Bar = 50 μm.

The fungal cell wall is an important cellular border that regulates several transport mechanisms, cellular metabolism, and connections with the extracellular environment. Its mechanical strength allows cells to tolerate turgor pressure, resulting in inhibition of cell lysis (Reinoso-Martin et al., 2003). After treatment with KC81 SUP, many swollen *M. oryzae* mycelia and germ tubes were observed. CFW staining was performed on mycelia treated with KC81 SUP. Fluorescence aggregated in the swollen *M. oryzae* mycelia (**Figure 3**), demonstrating that treatment with KC81 SUP perturbed the cell wall of *M. oryzae* Guy11.

### KC81 SUP disrupts actin cytoskeleton in *M. oryzae*

**Figure 4:**
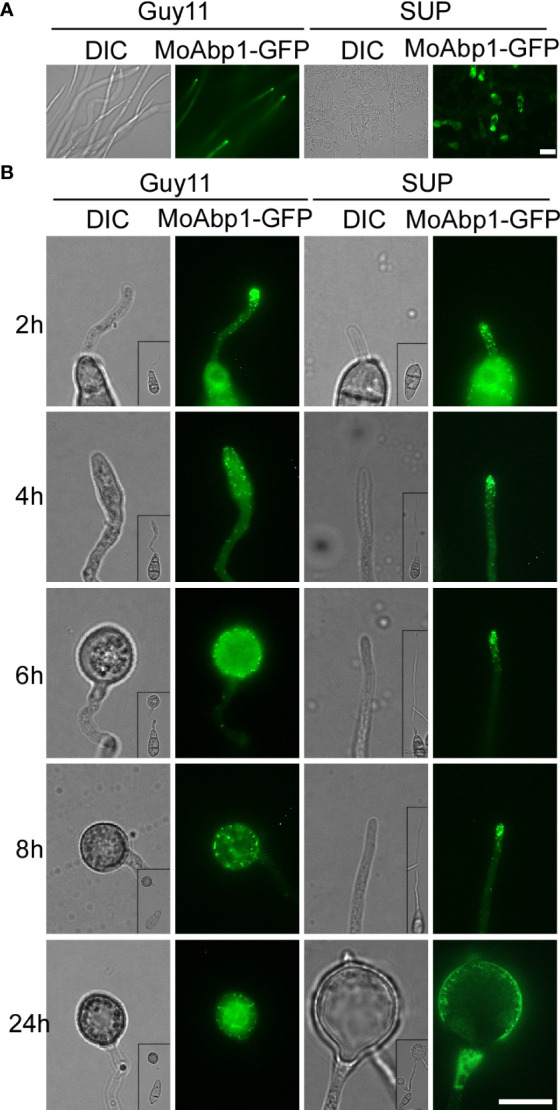
KC81 SUP impairs actin cytoskeleton organization of *M. oryzae*. **(A)** Live cell imaging experiment to show MoAbp1-GFP (green fluorescent protein) localization in *M. oryzae* mycelium in the presence or absence of KC81 SUP. **(B)** Live cell imaging experiment to show MoAbp1-GFP localization during *M. oryzae* appressorium development in the presence or absence of KC81 SUP. Bar = 10 μm.

The actin cytoskeleton plays an important role in the growth, development, and pathogenicity of *M. oryzae* (Dagdas et al., 2012; Li et al., 2017). To test whether KC81 SUP affects the actin cytoskeleton, we observed the actin cytoskeleton of Guy11 cells expressing the actin reporter, actin-binding protein MoAbp1-GFP. In the control, the GFP signal was observed in the apical membrane of hyphae and germ tubes and formed apical cortical patches. With appressorium development, the GFP signal moved along the cortex, away from the germ tube tip, and the GFP signal finally appeared in the center and cortical region of the appressoria and formed a ring in the center of the appressoria. In strains exposed to KC81 SUP, the GFP signal decreased sharply and only appeared in the cortex and cytoplasm of a few cells. The GFP signal could not move along the cortex away from the germ tube tip. It finally assembled in the cortex of swollen cells, and could not form a ring (**Figure 4**). These results suggest that KC81 SUP disrupts the actin cytoskeleton in *M. oryzae.*

### KC81 SUP impairs microtubule organization and mitosis in *M. oryzae*

**Figure 5:**
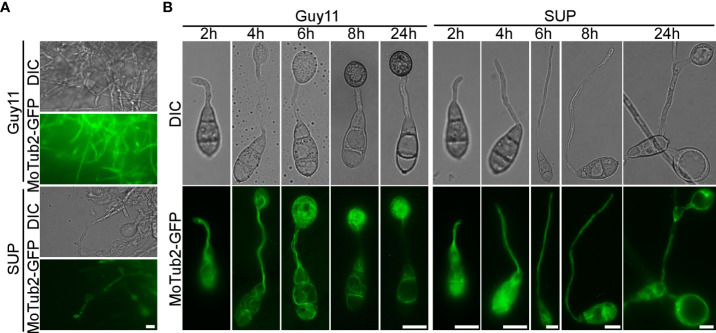
KC81 SUP impairs microtubule cytoskeleton organization of *M. oryzae*. **(A)** Live cell imaging experiment to show MoTub2-GFP localization in *M. oryzae* mycelium in the presence or absence of KC81 SUP. **(B)** Live cell imaging experiment to show MoTub2-GFP localization during *M. oryzae* appressorium development in the presence or absence of KC81 SUP. Bar = 10 μm.

**Figure 6:**
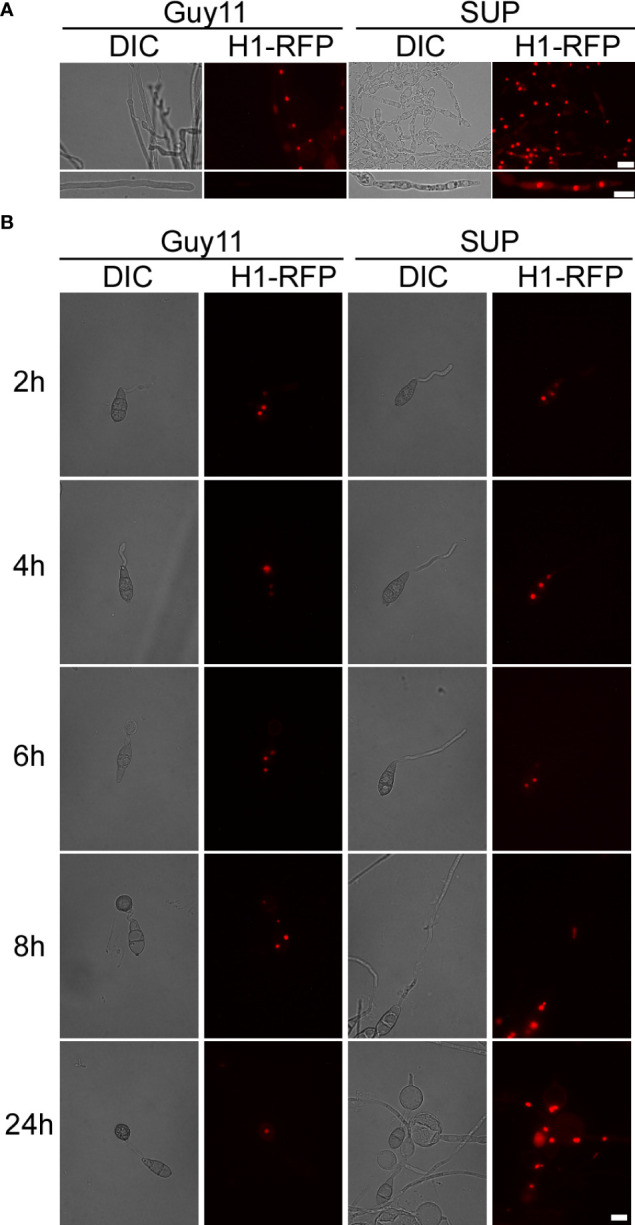
KC81 SUP impairs mitosis of *M. oryzae*. **(A)** Live cell imaging experiment to show MoH1-RFP (red fluorescent protein) localization in *M. oryzae* mycelium in the presence or absence of KC81 SUP. **(B)** Live cell imaging experiment to show MoH1-RFP localization during *M. oryzae* appressorium development in the presence or absence of KC81 SUP. Bar = 10 μm.

To cause rice blast disease, *M. oryzae* must properly organize microtubules and position nuclei during appressorium development (Saunders et al., 2010). To investigate the effects of KC81 SUP on nuclear division, migration, and microtubules during mycelial growth and appressorium formation, we observed SUP-treated *M. oryzae* expressing a histone H1-red fluorescent protein (H1-RFP) and β-tubulin-green fluorescent protein (MoTub2-GFP), as shown in **Figures 5**, **6**. Filamentous cross-linked green fluorescence was observed in untreated *M. oryzae* mycelia. Green fluorescence was observed only in a small number of mycelial cells and weakened or even disappeared in the treated strain (**Figure 5A**). Following spore germination, a polarized germ tube was formed and microtubules were aligned parallel to its longitudinal axis. When the conidia had germinated for 4 h, fluorescence aggregated to the sub-tip of the germ tube and formed a crossed network. With the development and maturation of the appressorium, the green fluorescence in conidia and germ tubes gradually moved into the appressorium, forming a cage-like arrangement around the nuclei. However, when treated with SUP, the green fluorescence signal did not accumulate in the sub-tips to form a crossed network. Finally, the green fluorescence of MoTub2-GFP was distributed in the conidia, long germ tubes, and cytoplasm of the swollen cells (**Figure 5B**).

In the control, a dot-like red fluorescent signal and a relatively weak elliptical red fluorescence signal, indicating that the nucleus is active, were observed in mature and tip cells of the mycelium, respectively. In the SUP-treated strain, the red fluorescence signal was strong and relatively round in both mature and tip cells of the hypha (**Figure 6A**). During spore germination of the control strain, one daughter nucleus migrated to the initial appressorium in the tube tip, whereas the other returned to the original conidial cell (8h). The germ tube tip swelled before mitosis and continued to develop, increasing in diameter upon receipt of the daughter nuclei. The three nuclei that remained in the spore were then broken down, with a single nucleus remaining in the appressorium. However, after SUP treatment, the germ tube tip did not form an initial appressorium over time to receive a new nucleus. SUP-treated conidia retained three nuclei and instead formed undifferentiated germlings with swollen cells (**Figure 6B**). These results indicate that KC81 SUP disturbs microtubule arrangement and mitosis in *M. oryzae*.

### KC81 SUP impairs autophagy in *M. oryzae*

**Figure 7:**
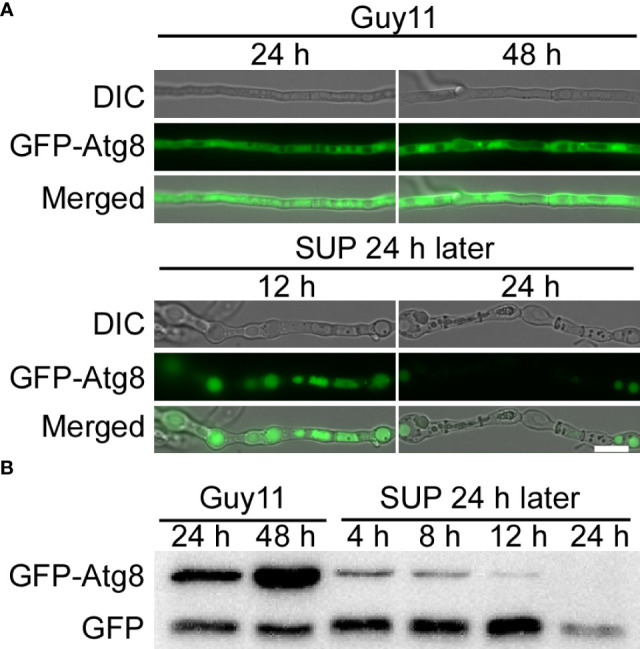
KC81 SUP promotes cell autophagy of *M. oryzae*. **(A)** Live cell imaging experiment to show GFP-Atg8 localization in *M. oryzae* mycelium in the presence or absence of KC81 SUP. **(B)** Immunoblotting was performed with anti-GFP antibodies. The extent of autophagy was estimated by calculating the amount of free GFP compared with the total amount of intact GFP-Atg8 and free GFP. Bar = 10 μm.

Autophagy, a stress response, plays a key role in the survival of eukaryotes (Kroemer et al., 2010; Mizushima and Komatsu, 2011). To study the effects of KC81 SUP on the autophagy process of *M. oryzae*, we observed the degradation of GFP-Atg8 in strain Guy11 following SUP treatment. The green fluorescence signal was observed mainly in the cytoplasm and rarely in vacuoles. The green fluorescence signal was present in vacuoles at 12h after SUP treatment. After SUP treatment for 24 h, the green fluorescence became very weak (**Figure 7A**). Furthermore, the level of autophagy was monitored by assessing GFP-Atg8 levels using western blotting. The degradation of GFP-ATG8 was significantly intensified by SUP treatment. GFP-ATG8 was almost completely degraded to GFP. However, the GFP band at 24 h after SUP treatment was very weak (**Figure 7B**), indicating that SUP might destroy *M. oryzae* cells and cause intracellular cytoplasmic outflow. Therefore, autophagy in *M. oryzae* is accelerated by SUP treatment.

### KC81 SUP impairs the localization of cell wall integrity and stress response component MoWsc1 in *M. oryzae*

**Figure 8:**
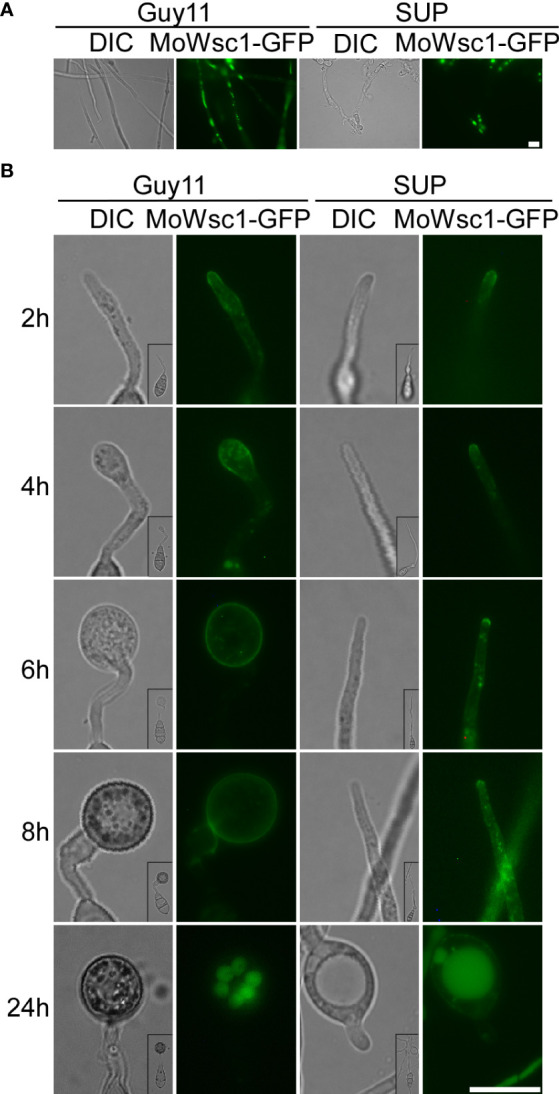
Cell wall integrity signaling pathway response proteins MoWsc1 respond to KC81 SUP. **(A)** Live cell imaging experiment to show MoWsc1-GFP localization in *M. oryzae* mycelium in the presence or absence of KC81 SUP. **(B)** Live cell imaging experiment to show MoWsc1-GFP localization during *M. oryzae* appressorium development in the presence or absence of KC81 SUP. Bar = 10 μm.

The cell wall integrity (CWI) and stress response protein Wsc1p acts as a dedicated sensor to initiate protective responses through an activated rescue pathway for *de novo* cell wall synthesis against external cell wall damage (Reinoso-Martin et al., 2003). In the control strain, Wsc1-GFP displayed a mostly polarized cell surface distribution along with some intracellular localization in mycelial cells with vigorous growth and changed its distribution to accumulate in the vacuole in mature cells. In the SUP-treated strains, Wsc1-GFP mainly accumulated in the vacuoles and plasma membranes of a few cells (**Figure 8A**). During the development of the appressorium, Wsc1-GFP was localized on the plasma membrane at the tip of the germ tube and transferred to the sub-tip plasma membrane along with some intracellular localization during the initial appressorium formation. With the development and maturation of the appressorium, internalized Wsc1-GFP increased and finally localized in the vacuoles of the appressorium. In the SUP-treated strain, Wsc1-GFP was localized on the plasma membrane at the tip of the germ tube and was continuously internalized but did not move to the sub-tip. Finally, even though much of the Wsc1-GFP was internalized into the vacuoles of swollen cells, a large number of GFP signals were still distributed on the plasma membrane (**Figure 8B**). These results suggest that *M. oryzae* may require Wsc1 to continuously sense stress caused by SUP.

### KC81 SUP activates phosphorylation of Mps1- and Pmk1- MAPK and MoCdc2

**Figure 9:**
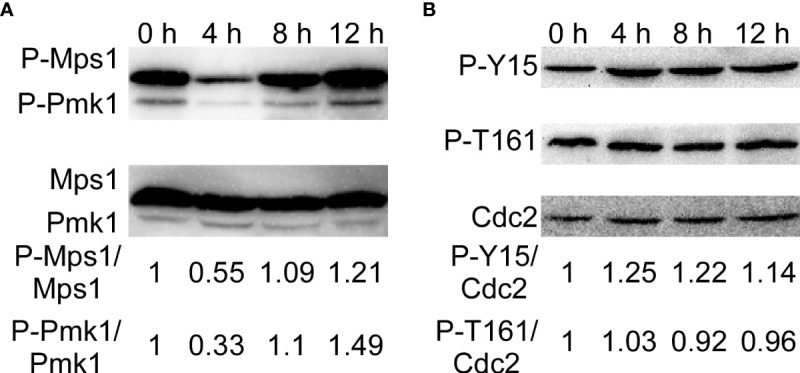
KC81 SUP activates phosphorylation of Mps1-, Pmk1- MAPK, and MoCdc2. **(A)** Western blot assays for phosphorylation of Mps1 and Pmk1. **(B)** Western blot assays for phosphorylation of Cdc2.

The CWI pathway is one of the most critical signaling mechanisms in response to adaptation to diverse environmental conditions in *M. oryzae* (Yin et al., 2016; Yin et al., 2020). Moreover, Wsc1 was continuously localized in the plasma membrane of the treated strains. Therefore, we determined the activity of the MAPK signaling pathways by detecting the phosphorylation of Mps1 and Pmk1. We found that phosphorylation of Mps1 and Pmk1 decreased after SUP treatment, but increased significantly after 8 and 12 h (**Figure 9A**). These results suggest that the CWI signaling pathway in *M. oryzae* is activated during the later stages of SUP treatment.

Mitosis in eukaryotic cells is regulated by Cdc2 kinase activation, including phosphorylation of Cdc2 at the Thr 161 site and dephosphorylation of Cdc2 at the Tyr 15 site. To further study the reasons for the mitotic changes in *M. oryzae* after SUP treatment, we detected the phosphorylation of Cdc2. After SUP treatment, there was no significant change in the phosphorylation of Cdc2 at Thr 161, but phosphorylation was significantly enhanced at Cdc2 Tyr 15 (**Figure 9B**). These results indicate that SUP inhibits mitosis in *M. oryzae*.

## Discussion

*M. oryzae* is one of the most destructive crop pathogens worldwide, causing huge losses in rice harvest every year (Dean et al., 2012; Wilson, 2021). Therefore, successful control of rice blast disease caused by this fungus is necessary. *Bacillus* spp. are a potential biocontrol agent explored in many crop systems as it is a potent producer of bioactive compounds with multiple biological properties against plant pathogenic fungi (Singh, 2018; Saxena et al., 2020). Biocontrol microbes can interact with plant pathogens through a variety of mechanisms, including the production of antibiotics and lytic enzymes, competition for nutrients and ecological niches, detoxification of toxins, and degradation of virulence factors (Schulz et al., 2006). *Bacillus* spp. produce many metabolites and destroy the cells of plant pathogenic fungi (Fira et al., 2018).

Previous intensive study of the infection mechanism of *M. oryzae* makes it an excellent model for understanding how the KC81 SUP might interact with phytopathogenic fungi in the context of interference with plant infection. First, we demonstrated that *B. subtilis* and SUP can inhibit the growth of *M. oryzae* and its ability to cause rice blast disease. We also showed that the SUP of *B. subtilis* KC81 not only inhibits functional appressorium formation and penetrant activity but also affects invasive fungal growth. Appressorium formation of *M. oryzae* exposed to SUP decreased significantly on both hydrophobic slides and rice leaf surfaces. In barley bioassays, SUP inhibited penetration peg formation and invasive hyphal development. Some of our findings are consistent with the results of previous studies. *Bacillus methylotrophicus* strain BC79 culture filtrate caused malformations and showed very high biocontrol efficiency for *M. oryzae* (Shan et al., 2013). Sterilized culture filtrate of *Bacillus australimaris* CQ07 can delay and even inhibit the germination of conidia and prevent functional appressorium development *in vitro* and *in vivo* (Chen et al., 2019). The culture filtrates of *Bacillus subtilis* strain BJ-1 significantly inhibited the mycelial growth of *M. oryzae* P131 and resulted in the disintegration of mycelium and the induction of abnormally swollen germ tubes (He et al., 2019).

The fungal cell wall is an essential cellular boundary that regulates many transport processes, cellular metabolism, and communication with the external environment. Owing to its mechanical strength, it allows cells to withstand turgor pressure and, consequently, prevent cell lysis (Cid et al., 1995; Klis et al., 2002). In this study, many swollen *M. oryzae* mycelia and germ tubes were observed after treatment with KC81 SUP as it perturbed the cell wall of *M. oryzae* Guy11. The correct remodeling of the cell wall is essential for the growth and formation of functional appressoria in *M. oryzae*. In the process of appressorium formation, the cell wall is essential for maintaining turgor, transforming turgor to directional force, and facilitating penetration of the penetration peg into host cells, which are necessary for the appressorium to successfully invade host cells (Talbot, 2019). A similar phenomenon occurs in the interaction between *Aspergillus* and *Bacillus* spp. Culture filtrates of *B. subtilis* and *Bacillus amyloliquefaciens* can inhibit the growth of *Aspergillus parasiticus* and aflatoxin production by inducing changes in fungal cell wall chitin and glucan contents (Siahmoshteh et al., 2018).

Based on the previously observed changes in growth, pathogenicity, functional appressorium formation, and the cell wall, it is interesting to know what changes have taken place in the conserved structure and biological processes of rice blast fungus cells to deal with the poisoning caused by external biocontrol agents. In treated *M. oryzae*, the actin cytoskeleton cannot move to the sub-tips of hyphae and germ tubes, which is necessary for the polar growth of *M. oryzae.* The actin cytoskeleton also cannot assemble into rings in time during the development and maturation of the appressorium, which is necessary for the formation of a functional appressorium (Dagdas et al., 2012; Li et al., 2017). After SUP treatment, tubulin did not accumulate in the sub-tips to form a cross-network in the germ tube tip, which is necessary for nuclear migration during appressorium formation. To infect the plant host, *M. oryzae* must properly assemble the cytoskeleton and position the nucleus during appressorium formation (Saunders et al., 2010).

The process, from the development of the appressorium to the formation of an appressorium with invasive ability, is accompanied by a timely cell cycle and autophagy (Veneault-Fourrey et al., 2006; Saunders et al., 2010). The three nuclei that remained in the spore were then broken down, with a single nucleus remaining in the appressorium. However, after SUP treatment, the germ tube tip did not form an initial appressorium in time to receive a new nucleus. SUP-treated conidia retained three nuclei and instead formed undifferentiated germlings with swollen cells. The cell cycle in eukaryotic cells is regulated by Cdc2 kinase activation, including phosphorylation of Cdc2 at Thr 161 site and dephosphorylation of Cdc2 at the Tyr 15 site (Wang et al., 2004; Sgarlata and Perez-Martin, 2005; Liu et al., 2015). The phosphorylation of Cdc2 at Thr 161 did not change much after SUP treatment, whereas it was dramatically increased at Cdc2 Tyr 15. SUP suppresses *M. oryzae* mitosis. Cells face various stresses during growth and development. Autophagy is a degradation process in which cells decompose their components under stressful conditions to circulate macromolecules and provide energy (Kroemer et al., 2010; Mizushima and Komatsu, 2011; Yin et al., 2020). By monitoring vacuolar transport and subsequent degradation of GFP-Atg8, autophagic flux can be observed. The GFP-Atg8 present inside the autophagosome is cleaved, and the contents are exposed to vacuolar hydrolases after the autophagosome membrane is lysed. The intact GFP portion cut from GFP-Atg8 is relatively resistant to vacuolar protein hydrolysis and accumulates in the vacuoles (Cheong and Klionsky, 2008; Nair et al., 2011; Yin et al., 2020). In this study, the degradation of GFP-Atg8 was significantly intensified by SUP treatment, and GFP-Atg8 was almost completely degraded to GFP, indicating that the autophagy process in *M. oryzae* was accelerated to recycle macromolecules and provide energy under SUP stress. However, the GFP band at 24 h after SUP treatment was very weak, indicating that SUP might destroy *M. oryzae* cells and cause intracellular cytoplasmic outflow.

The CWI pathway is one of the most critical signaling mechanisms for the response and adaptation of *M. oryzae* to different environmental conditions (Yin et al., 2016; Yin et al., 2020). Cell wall damage is sensed to a large extent by Wsc1, which is a key sensor of the cell wall integrity pathway. The cell wall integrity and stress response protein Wsc1p, as a specific sensor, can initiate a protective response through the activated salvage pathway for nascent cell wall synthesis to withstand external cell wall destruction (Reinoso-Martin et al., 2003). In yeast, Wsc1 localizes to the periphery of cells, accumulates at synthesis sites of the cell wall, and is involved in the maintenance of CWI (Verna et al., 1997; Piao et al., 2007; Straede and Heinisch, 2007). Yeast Wsc1 also regulates the osmotic stress response by reorganizing the actin cytoskeleton and regulating the alkaline stress response by activating the MAPK cascade to mediate the expression of several pH-regulating genes (Raboy et al., 1999; Gualtieri et al., 2004; Serrano et al., 2006; Bermejo et al., 2010; Heinisch et al., 2010). As a dedicated sensor, Wsc1p senses cell wall damage caused by the antifungal drug caspofungin and initiates a protective response through activated salvage pathways to resynthesize the cell wall (Reinoso-Martin et al., 2003). In this study, Wsc1 was increased on lipid membranes in SUP-treated *M. oryzae*, especially at the tips of the active hyphae and germ tubes. Furthermore, we evaluated the activation of the MAPK signaling pathways by analyzing the phosphorylation of Mps1 and Pmk1. We discovered that the phosphorylation of Mps1 and Pmk1 was reduced following SUP treatment, but increased considerably after 8 and 12 h. The integrity of the cell wall might have been destroyed at 8 h, and *M. oryzae* needed to start the corresponding response. *M. oryzae* may require Wsc1 to continuously sense the destructive stress from SUP and activate the CWI pathway, further triggering a series of intracellular responses. We tried to explore the point at which KC81 destroys *M. oryzae* cells and the point at which *M. oryzae* cells respond significantly to this destruction. However, we have found this to be difficult because the state of a single hypha differed from that of another even in the same flask, and the action of SUP is a relatively slow integrated process.

Multiple signaling pathways and cell biological processes intersect in fungal cells in response to external stresses and induced signals. In particular, the importance of the CWI pathway in mediating the response of *M. oryzae* to SUP led us to examine the cellular biological process of cross-talking with the CWI signaling pathway. This is a well-known mediator in response to cell wall stress and is associated with osmotic stress adaptation and drug treatments (Reinoso-Martin et al., 2003). We found that SUP may exert its toxicity towards *M. oryzae* through multiple signaling pathways and cellular processes. The CWI signaling pathway and cell cycle progression cross-talk govern and coordinate cellular growth, development, and pathogenicity in response to cell stress in fungi (Shiozaki and Russell, 1995; Feng et al., 2021). The CWI signaling pathway also interacts with autophagy in yeast and *M. oryzae* in response to stress (Yin et al., 2020; Oses-Ruiz et al., 2021). The process of cell cycle regulation in the formation of functional appressorium is closely followed by autophagy, cell wall remodeling, and cytoskeleton remodeling. The autophagy process ensures that spores are degraded and recycled into the appressorium to form turgor (Veneault-Fourrey et al., 2006; Kershaw and Talbot, 2009; Oses-Ruiz et al., 2017; Eseola et al., 2021). An intact cell wall and remodeled cytoskeleton are essential for maintaining turgor and converting turgor pressure into directed mechanical forces to penetrate host cells (Dagdas et al., 2012). At the same time, when the MAPK signaling pathway is disrupted, the cytoskeleton assembly in *M. oryzae* is also abnormal and *M. oryzae* cannot effectively infect the host (Dagdas et al., 2012; Sakulkoo et al., 2018; Oses-Ruiz et al., 2021). The disruption of these cell signaling pathways and cell biological processes by SUP will inevitably interfere with the growth, development, and pathogenicity of *M. oryzae*. The activation of the CWI signaling pathway, the acceleration of cytoplasmic autophagy, and the slowing of the cell cycle in *M. oryzae* are manifestations of resistance to external adverse environments.

*Bacillus* spp. is potentially very effective for controlling rice blast because it prevents leaf infection at a very early stage, before cuticle penetration. Furthermore, since *Bacillus* spp. may act on multiple cross-linked signaling pathways and cellular processes that regulate the growth and pathogenicity of *M. oryzae*, the chances of selection for specific resistant *M. oryzae* mutants are likely to be low. In conclusion, the intracellular response changes of *M. oryzae* during intoxication by *B. subtilis* are the molecular ecological basis for understanding the interaction between biocontrol bacteria and fungi, and *Bacillus* may constitute a possible natural biocontrol for rice blast.

## Data availability statement

The original contributions presented in the study are included in the article/Supplementary Material. Further inquiries can be directed to the corresponding author.

## Author contributions

LWL, JHJ, and YRL conceived and designed the study. YRL, KLL, RRC, and LWL performed the experiments. LWL and YRL analyzed the experimental data. LWL and JHJ contributed reagents, materials, and analytical tools. LWL and YRL wrote the manuscript. All the authors have read and approved the final manuscript.

## Funding

This research was supported by the National Natural Science Foundation of China (NSFC31901831 to LL), the Natural Science Foundation of Jiangsu Province (BK20181005 to LL), the Natural Science Foundation of the Jiangsu Higher Education Institutions of China (18KJB210004 to LL), the State Key Laboratory of Ecological Pest Control for Fujian and Taiwan Crops (SKL2019002 to LL), and a Project Funded by the Priority Academic Program Development of Jiangsu Higher Education Institutions.

## Conflict of interest

The authors declare that the research was conducted in the absence of any commercial or financial relationships that could be construed as a potential conflict of interest.

## Publisher’s note

All claims expressed in this article are solely those of the authors and do not necessarily represent those of their affiliated organizations, or those of the publisher, the editors and the reviewers. Any product that may be evaluated in this article, or claim that may be made by its manufacturer, is not guaranteed or endorsed by the publisher.

## References

[B1] Bermejo C. Garcia R. Straede A. Rodriguez-Pena J. M. Nombela C. Heinisch J. J.. (2010). Characterization of sensor-specific stress response by transcriptional profiling of wsc1 and mid2 deletion strains and chimeric sensors in *Saccharomyces cerevisiae* . Omics-a. J. Integr. Biol. 14, 679–688. doi: 10.1089/omi.2010.0060 20958245

[B2] Cansado J. Soto T. Franco A. Vicente-Soler J. Madrid M. (2022). The fission yeast cell integrity pathway: a functional hub for cell survival upon stress and beyond. J. Fungi. 8, 32. doi: 10.3390/jof8010032 PMC8781887 35049972

[B3] Chen W. Zhao L. Li H. Dong Y. Xu H. Guan Y.. (2019). The isolation of the antagonistic strain *Bacillus australimaris* CQ07 and the exploration of the pathogenic inhibition mechanism of *Magnaporthe oryzae* . PloS One 14, e0220410. doi: 10.1371/journal.pone.0220410 31404061 PMC6690535

[B4] Cheong H. Klionsky D. J. (2008). Biochemical methods to monitor autophagy-related processes in yeast. Methods Enzymol. 451, 1–26. doi: 10.1016/S0076-6879(08)03201-1 19185709

[B5] Cid V. J. Duran A. Del Rey F. Snyder M. P. Nombela C. Sanchez M. (1995). Molecular basis of cell integrity and morphogenesis in *Saccharomyces cerevisiae* . Microbiol. Rev. 59, 345–386. doi: 10.1128/mr.59.3.345-386.1995 7565410 PMC239365

[B6] Dagdas Y. F. Yoshino K. Dagdas G. Ryder L. S. Bielska E. Steinberg G.. (2012). Septin-mediated plant cell invasion by the rice blast fungus, *Magnaporthe oryzae* . Science 336, 1590–1595. doi: 10.1126/science.1222934 22723425

[B7] Dean R. Van Kan J. A. Pretorius Z. A. Hammond-Kosack K. E. Di Pietro A. Spanu P. D.. (2012). The top 10 fungal pathogens in molecular plant pathology. Mol. Plant Pathol. 13, 414–430. doi: 10.1111/j.1364-3703.2011.00783.x 22471698 PMC6638784

[B8] Eseola A. B. Ryder L. S. Oses-Ruiz M. Findlay K. Yan X. Cruz-Mireles N.. (2021). Investigating the cell and developmental biology of plant infection by the rice blast fungus *Magnaporthe oryzae* . Fungal Genet. Biol. 154, 103562. doi: 10.1016/j.fgb.2021.103562 33882359

[B9] Feng W. Yin Z. Wu H. Liu P. Liu X. Liu M.. (2021). Balancing of the mitotic exit network and cell wall integrity signaling governs the development and pathogenicity in *Magnaporthe oryzae* . PloS Pathog. 17, e1009080. doi: 10.1371/journal.ppat.1009080 33411855 PMC7817018

[B10] Fira D. Dimkic I. Beric T. Lozo J. Stankovic S. (2018). Biological control of plant pathogens by bacillus species. J. Biotechnol. 285, 44–55. doi: 10.1016/j.jbiotec.2018.07.044 30172784

[B11] George V. T. Brooks G. Humphrey T. C. (2007). Regulation of cell cycle and stress responses to hydrostatic pressure in fission yeast. Mol. Biol. Cell 18, 4168–4179. doi: 10.1091/mbc.e06-12-1141 17699598 PMC1995737

[B12] Gualtieri T. Ragni E. Mizzi L. Fascio U. Popolo L. (2004). The cell wall sensor Wsc1p is involved in reorganization of actin cytoskeleton in response to hypo-osmotic shock in *Saccharomyces cerevisiae* . Yeast 21, 1107–1120. doi: 10.1002/yea.1155 15484288

[B13] Heinisch J. J. Dupres V. Wilk S. Jendretzki A. Dufrene Y. F. (2010). Single-molecule atomic force microscopy reveals clustering of the yeast plasma-membrane sensor Wsc1. PloS One 5, e11104. doi: 10.1371/journal.pone.0011104 20559440 PMC2885430

[B14] He Y. W. Zhu M. L. Huang J. B. Hsiang T. Zheng L. (2019). Biocontrol potential of a *Bacillus subtilis* strain BJ-1 against the rice blast fungus *Magnaporthe oryzae* . Can. J. Plant Pathol. 41, 47–59. doi: 10.1080/07060661.2018.1564792

[B15] Huang W. Liu X. Zhou X. Wang X. Liu X. Liu H. (2020). Calcium signaling is suppressed in *Magnaporthe oryzae* conidia by *Bacillus cereus* HS24. Phytopathology 110, 309–316. doi: 10.1094/PHYTO-08-18-0311-R 31556343

[B16] Jeon J. Goh J. Yoo S. Chi M. H. Choi J. Rho H. S.. (2008). A putative MAP kinase kinase kinase, MCK1, is required for cell wall integrity and pathogenicity of the rice blast fungus, *Magnaporthe oryzae* . Mol. Plant Microbe Interact. 21, 525–534. doi: 10.1094/MPMI-21-5-0525 18393612

[B17] Kagami Y. Yoshida K. (2016). The functional role for condensin in the regulation of chromosomal organization during the cell cycle. Cell Mol. Life Sci. 73, 4591–4598. doi: 10.1007/s00018-016-2305-z 27402120 PMC11108269

[B18] Karthikeyan V. Gnanamanickam S. S. (2008). Biological control of setaria blast (*Magnaporthe grisea*) with bacterial strains. Crop Prot. 27, 263–267. doi: 10.1016/j.cropro.2007.05.013

[B19] Kershaw M. J. Talbot N. J. (2009). Genome-wide functional analysis reveals that infection-associated fungal autophagy is necessary for rice blast disease. Proc. Natl. Acad. Sci. U.S.A. 106, 15967–15972. doi: 10.1073/pnas.0901477106 19717456 PMC2747227

[B20] Klis F. M. Mol P. Hellingwerf K. Brul S. (2002). Dynamics of cell wall structure in *Saccharomyces cerevisiae* . FEMS Microbiol. Rev. 26, 239–256. doi: 10.1111/j.1574-6976.2002.tb00613.x 12165426

[B21] Kock C. Arlt H. Ungermann C. Heinisch J. J. (2016). Yeast cell wall integrity sensors form specific plasma membrane microdomains important for signalling. Cell Microbiol. 18, 1251–1267. doi: 10.1111/cmi.12635 27337501

[B22] Kroemer G. Marino G. Levine B. (2010). Autophagy and the integrated stress response. Mol. Cell 40, 280–293. doi: 10.1016/j.molcel.2010.09.023 20965422 PMC3127250

[B23] Latge J. P. (2007). The cell wall: a carbohydrate armour for the fungal cell. Mol. Microbiol. 66, 279–290. doi: 10.1111/j.1365-2958.2007.05872.x 17854405

[B24] Levin D. E. (2011). Regulation of cell wall biogenesis in *Saccharomyces cerevisiae*: the cell wall integrity signaling pathway. Genetics 189, 1145–1175. doi: 10.1534/genetics.111.128264 22174182 PMC3241422

[B25] Li L. Chen X. Zhang S. Yang J. Chen D. Liu M.. (2017). MoCAP proteins regulated by MoArk1-mediated phosphorylation coordinate endocytosis and actin dynamics to govern development and virulence of *Magnaporthe oryzae* . PloS Genet. 13, e1006814. doi: 10.1371/journal.pgen.1006814 28542408 PMC5466339

[B26] Li L. Li D. Zhang L. Wang J. Yang X. Jiang J. (2018). Isolation and identification of garlic endophytes and screening of their antagonistic strains. Jiangsu. Agric. Sci. 46, 97–101. doi: 10.15889/j.issn.1002-1302.2018.05.026

[B27] Liu X. Y. Bao T. T. Zheng L. Kgosi V. T. Liu X. Y. Liu H. X. (2021). Cell wall integrity in *Magnaporthe oryzae* is weakened by proteins secreted by *Bacillus licheniformis* BL06. Biol. Control. 157, 104582. doi: 10.1016/j.biocontrol.2021.104582

[B28] Liu J. Kipreos E. T. (2000). Evolution of cyclin-dependent kinases (CDKs) and CDK-activating kinases (CAKs): differential conservation of CAKs in yeast and metazoa. Mol. Biol. Evol. 17, 1061–1074. doi: 10.1093/oxfordjournals.molbev.a026387 10889219

[B29] Liu H. Zhang S. Ma J. Dai Y. Li C. Lyu X.. (2015). Two Cdc2 kinase genes with distinct functions in vegetative and infectious hyphae in *Fusarium graminearum* . PloS Pathog. 11, e1004913. doi: 10.1371/journal.ppat.1004913 26083253 PMC4470668

[B30] Mendenhall M. D. Hodge A. E. (1998). Regulation of Cdc28 cyclin-dependent protein kinase activity during the cell cycle of the yeast *Saccharomyces cerevisiae* . Microbiol. Mol. Biol. Rev. 62, 1191–1243. doi: 10.1128/MMBR.62.4.1191-1243.1998 9841670 PMC98944

[B31] Mizushima N. Komatsu M. (2011). Autophagy: renovation of cells and tissues. Cell 147, 728–741. doi: 10.1016/j.cell.2011.10.026 22078875

[B32] Nair U. Thumm M. Klionsky D. J. Krick R. (2011). GFP-Atg8 protease protection as a tool to monitor autophagosome biogenesis. Autophagy 7, 1546–1550. doi: 10.4161/auto.7.12.18424 22108003 PMC3327617

[B33] Oses-Ruiz M. Cruz-Mireles N. Martin-Urdiroz M. Soanes D. M. Eseola A. B. Tang B.. (2021). Appressorium-mediated plant infection by *Magnaporthe oryzae* is regulated by a Pmk1-dependent hierarchical transcriptional network. Nat. Microbiol. 6, 1383–1397. doi: 10.1038/s41564-021-00978-w 34707224

[B34] Oses-Ruiz M. Sakulkoo W. Littlejohn G. R. Martin-Urdiroz M. Talbot N. J. (2017). Two independent s-phase checkpoints regulate appressorium-mediated plant infection by the rice blast fungus *Magnaporthe oryzae* . Proc. Natl. Acad. Sci. U.S.A. 114, E237–E244. doi: 10.1073/pnas.1611307114 28028232 PMC5240714

[B35] Petersen J. Hagan I. M. (2005). Polo kinase links the stress pathway to cell cycle control and tip growth in fission yeast. Nature 435, 507–512. doi: 10.1038/nature03590 15917811

[B36] Piao H. L. Machado I. M. P. Payne G. S. (2007). NPFXD-mediated endocytosis and function of a yeast cell is required for polarity wall stress sensor. Mol. Biol. Cell 18, 57–65. doi: 10.1091/mbc.e06-08-0721 17065552 PMC1751320

[B37] Raboy B. Marom A. Dor Y. Kulka R. G. (1999). Heat-induced cell cycle arrest of *Saccharomyces cerevisiae*: involvement of the *RAD6*/*UBC2* and *WSC2* genes in its reversal. Mol. Microbiol. 32, 729–739. doi: 10.1046/j.1365-2958.1999.01389.x 10361277

[B38] Reinoso-Martin C. Schuller C. Schuetzer-Muehlbauer M. Kuchler K. (2003). The yeast protein kinase c cell integrity pathway mediates tolerance to the antifungal drug caspofungin through activation of Slt2p mitogen-activated protein kinase signaling. Eukaryot. Cell 2, 1200–1210. doi: 10.1128/EC.2.6.1200-1210.2003 14665455 PMC326656

[B39] Rong S. Xu H. Li L. Chen R. Gao X. Xu Z. (2020). Antifungal activity of endophytic *Bacillus safensis* B21 and its potential application as a biopesticide to control rice blast. Pestic. Biochem. Physiol. 162, 69–77. doi: 10.1016/j.pestbp.2019.09.003 31836057

[B40] Sakulkoo W. Oses-Ruiz M. Oliveira Garcia E. Soanes D. M. Littlejohn G. R. Hacker C.. (2018). A single fungal MAP kinase controls plant cell-to-cell invasion by the rice blast fungus. Science 359, 1399–1403. doi: 10.1126/science.aaq0892 29567712

[B41] Saunders D. G. Aves S. J. Talbot N. J. (2010). Cell cycle-mediated regulation of plant infection by the rice blast fungus. Plant Cell 22, 497–507. doi: 10.1105/tpc.109.072447 20190078 PMC2845407

[B42] Saxena A. K. Kumar M. Chakdar H. Anuroopa N. Bagyaraj D. J. (2020). *Bacillus* species in soil as a natural resource for plant health and nutrition. J. Appl. Microbiol. 128, 1583–1594. doi: 10.1111/jam.14506 31705597

[B43] Schulz B. J. E. Boyle C. J. C. Sieber T. N. (2006). Microbial root endophytes (Berlin: Springer).

[B44] Serrano R. Martin H. Casamayor A. Arino J. (2006). Signaling alkaline pH stress in the yeast *Saccharomyces cerevisiae* through the Wsc1 cell surface sensor and the Slt2 MAPK pathway. J. Biol. Chem. 281, 39785–39795. doi: 10.1074/jbc.M604497200 17088254

[B45] Sgarlata C. Perez-Martin J. (2005). Inhibitory phosphorylation of a mitotic cyclin-dependent kinase regulates the morphogenesis, cell size and virulence of the smut fungus *Ustilago maydis* . J. Cell Sci. 118, 3607–3622. doi: 10.1242/jcs.02499 16046476

[B46] Shan H. Y. Zhao M. M. Chen D. X. Cheng J. L. Li J. Feng Z. Z.. (2013). Biocontrol of rice blast by the phenaminomethylacetic acid producer of *Bacillus methylotrophicus* strain BC79. Crop Prot. 44, 29–37. doi: 10.1016/j.cropro.2012.10.012

[B47] Shiozaki K. Russell P. (1995). Cell-cycle control linked to extracellular environment by map kinase pathway in fission yeast. Nature 378, 739–743. doi: 10.1038/378739a0 7501024

[B48] Siahmoshteh F. Hamidi-Esfahani Z. Spadaro D. Shams-Ghahfarokhi M. Razzaghi-Abyaneh M. (2018). Unraveling the mode of antifungal action of *Bacillus subtilis* and *Bacillus amyloliquefaciens* as potential biocontrol agents against aflatoxigenic *Aspergillus parasiticus* . Food Control. 89, 300–307. doi: 10.1016/j.foodcont.2017.11.010

[B49] Singh V. M. (2018). Role of rhizospheric microbes in soil (New York, NY: Springer Berlin Heidelberg).

[B50] Spence C. A. Raman V. Donofrio N. M. Bais H. P. (2014). Global gene expression in rice blast pathogen *Magnaporthe oryzae* treated with a natural rice soil isolate. Planta 239, 171–185. doi: 10.1007/s00425-013-1974-1 24126723

[B51] Straede A. Heinisch J. J. (2007). Functional analyses of the extra- and intracellular domains of the yeast cell wall integrity sensors Mid2 and Wsc1. FEBS Lett. 581, 4495–4500. doi: 10.1016/j.febslet.2007.08.027 17761172

[B52] Talbot N. J. (2019). Appressoria. Curr. Biol. 29, R144–R146. doi: 10.1016/j.cub.2018.12.050 30836078

[B53] Veneault-Fourrey C. Barooah M. Egan M. Wakley G. Talbot N. J. (2006). Autophagic fungal cell death is necessary for infection by the rice blast fungus. Science 312, 580–583. doi: 10.1126/science.1124550 16645096

[B54] Verna J. Lodder A. Lee K. Vagts A. Ballester R. (1997). A family of genes required for maintenance of cell wall integrity and for the stress response in *Saccharomyces cerevisiae* . Proc. Natl. Acad. Sci. U.S.A. 94, 13804–13809. doi: 10.1073/pnas.94.25.13804 9391108 PMC28388

[B55] Wang H. Gari E. Verges E. Gallego C. Aldea M. (2004). Recruitment of Cdc28 by Whi3 restricts nuclear accumulation of the G1 cyclin-cdk complex to late G1. EMBO J. 23, 180–190. doi: 10.1038/sj.emboj.7600022 14685274 PMC1271660

[B56] Wang J. Xu C. Sun Q. Xu J. Chai Y. Berg G.. (2021). Post-translational regulation of autophagy is involved in intra-microbiome suppression of fungal pathogens. Microbiome 9, 131. doi: 10.1186/s40168-021-01077-y 34092253 PMC8182927

[B57] Wilson R. A. (2021). Magnaporthe oryzae. Trends Microbiol. 29, 663–664. doi: 10.1016/j.tim.2021.03.019 33926783

[B58] Wu S. Liu G. Zhou S. Sha Z. Sun C. (2019). Characterization of antifungal lipopeptide biosurfactants produced by marine bacterium *Bacillus* sp. CS30. Mar. Drugs 17, 199. doi: 10.3390/md17040199 PMC6520760 30934847

[B59] Xu J. R. Staiger C. J. Hamer J. E. (1998). Inactivation of the mitogen-activated protein kinase Mps1 from the rice blast fungus prevents penetration of host cells but allows activation of plant defense responses. Proc. Natl. Acad. Sci. U.S.A. 95, 12713–12718. doi: 10.1073/pnas.95.21.12713 9770551 PMC22896

[B60] Yin Z. Feng W. Chen C. Xu J. Li Y. Yang L.. (2020). Shedding light on autophagy coordinating with cell wall integrity signaling to govern pathogenicity of *Magnaporthe oryzae* . Autophagy 16, 900–916. doi: 10.1080/15548627.2019.1644075 31313634 PMC7144863

[B61] Yin Z. Tang W. Wang J. Liu X. Yang L. Gao C.. (2016). Phosphodiesterase MoPdeH targets MoMck1 of the conserved mitogen-activated protein (MAP) kinase signalling pathway to regulate cell wall integrity in rice blast fungus *Magnaporthe oryzae* . Mol. Plant Pathol. 17, 654–668. doi: 10.1111/mpp.12317 27193947 PMC6638318

[B62] Zhang H. Liu K. Zhang X. Tang W. Wang J. Guo M.. (2011). Two phosphodiesterase genes, PDEL and PDEH, regulate development and pathogenicity by modulating intracellular cyclic AMP levels in *Magnaporthe oryzae* . PloS One 6, e17241. doi: 10.1371/journal.pone.0017241 21386978 PMC3046207

[B63] Zhang L. Sun C. (2018). Fengycins, cyclic lipopeptides from marine *Bacillus subtilis* strains, kill the plant-pathogenic fungus *Magnaporthe grisea* by inducing reactive oxygen species production and chromatin condensation. Appl. Environ. Microbiol. 84, e00445-18. doi: 10.1128/AEM.00445-18 29980550 PMC6122000

